# Metformin is associated with fewer major adverse cardiac events among patients with a new diagnosis of type 2 diabetes mellitus: A propensity score-matched nationwide study

**DOI:** 10.1097/MD.0000000000007507

**Published:** 2017-07-14

**Authors:** Kuang-Tso Lee, Yung-Hsin Yeh, Shang-Hung Chang, Lai-Chu See, Cheng-Hung Lee, Lung-Sheng Wu, Jia-Rou Liu, Chi-Tai Kuo, Ming-Shien Wen

**Affiliations:** a Chang Gung University and Department of Cardiology, Chang Gung Memorial Hospital, Taipei; b Department of Public Health, College of Medicine, Chang Gung University; c Biostatistics Core Laboratory, Molecular Medicine Research Center, Chang Gung University, Taiwan.

**Keywords:** diabetes mellitus, major adverse cardiac events, metformin

## Abstract

Early type 2 diabetes mellitus (DM) may only require lifestyle modifications for glycemic control without the need for oral hypoglycemic agents (OHAs). Metformin is believed to improve cardiovascular outcomes in patients with DM, and it is considered to be a first-line therapy. However, it is unclear whether metformin is beneficial for patients with a new diagnosis of DM compared to those who do not need OHAs for glycemic control.

Data were obtained from a population-based health care database in Taiwan. Patients with a new diagnosis of DM were enrolled if they received metformin monotherapy only between 1999 and 2010. A 4:1 propensity score-matched cohort of patients with a new diagnosis of DM who did not take OHAs or insulin during follow-up was also enrolled. The primary study endpoint was the occurrence of major adverse cardiovascular events (MACEs). The time to the endpoints was compared between groups using Cox proportional hazards models.

A total of 474,410 patients with DM were enrolled. During a mean 5.8 years of follow-up, the incidence of MACEs was 1.072% (1072 per 100,000 person-years) in the metformin monotherapy group versus 1.165% in the lifestyle modification group (those who did not take OHAs) (*P* < .001). After adjusting for confounders, metformin independently protected the DM patients from MACEs (hazard ratio: 0.83, *P* < .001). The metformin group also had an improved MACE-free survival profile from year 1 to year 12 (*P* < .001).

In addition to lifestyle modifications, the patients with a new diagnosis of DM treated with metformin monotherapy had a lower MACE rate than those who did not take OHAs. Our findings suggest that metformin may be given early to patients with a new diagnosis of DM, even when they do not need OHAs for glycemic control.

## 1 Introduction

The global prevalence of type 2 diabetes mellitus (DM) is 9% among adults, causing millions of deaths.^[1]^ The primary goal of DM treatment is to reduce the progression of cardiovascular complications through optimal glycemic control. Virtually all patients with early DM are advised to modify their lifestyle such as with appropriate diet control and exercise as initial glycemic control with or without oral hypoglycemic agents (OHAs). OHAs may not be administered for the patients in whom glycemic control is optimal after lifestyle modifications. Metformin is recommended as first-line therapy in patients without contraindications based on reports demonstrating the reduction of major adverse cardiovascular events (MACEs) in various diabetic populations, including patients with atherothrombosis, myocardial infarction, and heart failure, and patients who have undergone coronary interventions.^[2–12]^ Large trials conducted by the UK Prospective Diabetes Study have validated the safety profile of metformin for its early use in diabetic patients, and demonstrated the overall cost effectiveness.^[2,15,16]^ A recent trial also demonstrated that early metformin treatment improved the distribution of lipoprotein.^[17]^ However, in addition to lifestyle modifications, it is unclear whether metformin montherapy is beneficial for patients with a new diagnosis of DM compared to those who do not need OHAs for glycemic control. Therefore, we conducted this propensity score-matched nationwide study to investigate this issue.

## 2 Methods

### 2.1 Data sources and source population

The study sample was a dynamic population-based cohort enrolled from The Longitudinal Cohort of Diabetes Patients Database 1999–2010 (LHDB), a subset of all patients who received benefits from the universal Taiwan National Health Institutes (TNHI) program. Patients with a first diagnosis of DM between 1999 and 2010 were included if they: had at least 1 hospitalization with DM as one of the diagnoses (A-code A181 before 2000, or International Classification of Diseases, Ninth Revision, Clinical Modification [*ICD–9-CM*] code 250 after 2000), or had at least 2 outpatient visits owing to DM in the same calendar year, and had never been hospitalized or treated before 1999 as an outpatient owing to DM. The LHDB provides access to data for research purposes on no >10% of the population covered by the TNHI program. In the current analysis, 1.44 million eligible patients were randomly selected into the initial dataset. The LHDB includes data: after the diagnosis of DM, before the diagnosis of DM, and regarding treatment for diseases other than DM. The LHDB has been validated to be representative of all patients with DM in Taiwan.^[18,19]^ The identification number of each patient is encrypted as part of an established policy to protect privacy, and therefore informed consent was not required. This study was approved by the Institutional Review Board of the Chang Gung Medical Foundation, Taiwan.

### 2.2 Study population

**Figure 1:**
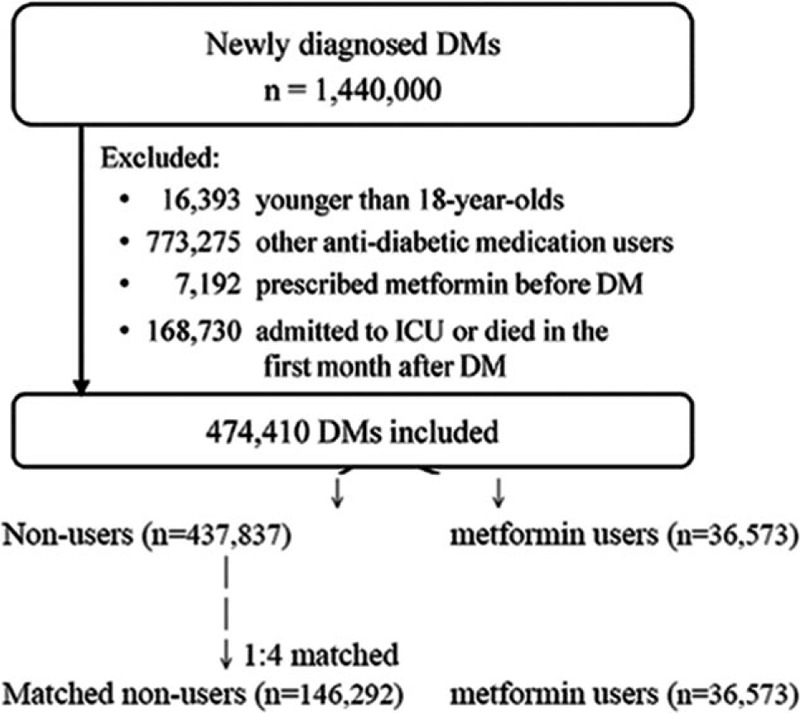
Study Flowchart. DM = diabetes mellitus, ICU = intensive care unit.

Using a new user design,^[20]^ we identified patients aged 18 years or older who used only metformin as their sole hypoglycemic agent from the LHDB described in 2.1. Patients with a new diagnosis of DM were included, and the exclusion criteria were: a prescription for metformin before the onset of DM, or a prescriptions for metformin for a total of less than 30 days, and patients admitted to an intensive care unit and those who died within the first month after the diagnosis of DM. The primary outcome was the onset of MACEs during the follow-up period. Follow-up was calculated as the time interval from the initial prescription of metformin to either the onset of MACEs, the date of death, or December 31, 2011, whichever came first (Fig. 1).

Subjects diagnosed with DM who did not take OHAs were classified into the lifestyle modification group because the official policy of TNHI requires that all physicians advise patients with DM to modify their lifestyle at every clinic visit.^[21]^ Among these patients, a 4:1 propensity score-matched cohort was selected by age, sex, years of diagnosis, co-morbidities, and medications and used as the reference group (Table 1). The patients were permitted to use statins or antihypertensive medications including angiotensin receptor blockers, angiotensin-converting enzyme inhibitors, beta blockers, and calcium channel blockers. Both the metformin monotherapy group and lifestyle modification group did not receive any other OHAs thorough the follow-up period.

**Table 1:**
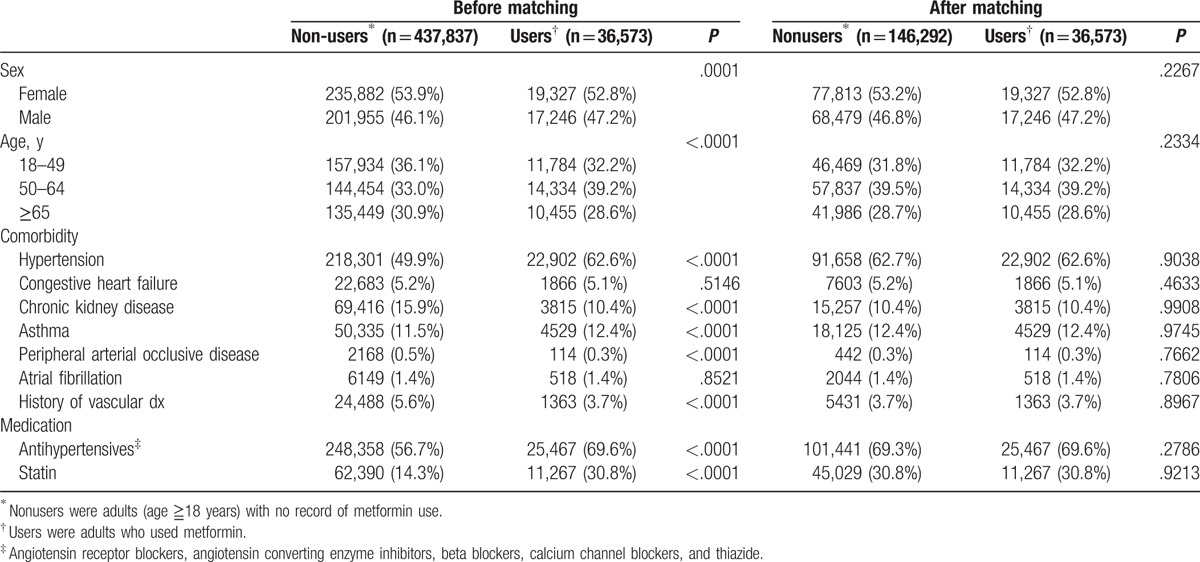
Demographic and comorbidity characteristics of the DM patients diagnosed by status of metformin use.

### 2.3 Ascertainment of MACEs

MACEs were defined as a diagnosed with one of the following ICD–9-CM codes: myocardial infarction (410), stroke (A290-A294 before 2000 or 430–437 after 2000), or one of the following Taiwan NHI procedure codes: percutaneous coronary intervention (33076A, 33076B, 33077A, 33077B, 33078A, 33078B), and coronary artery bypass graft (CABG) (68023A, 68023B, 68024A, 68024B, 68025A, 68025B). A previous MACE was defined as a hospitalization owing to a MACE before the index date, and a new-onset MACE was defined as a hospitalization owing to a MACE after the index date.

### 2.4 Ascertainment of comorbidities

The presence of either an A-code before 2000 or ICD-9-CM code after 2000 was used to determine the status of comorbid conditions, including hypertension (A26 or 401, 402), congestive heart failure (428), chronic kidney disease (A350, 580–589), asthma/chronic obstructive pulmonary disease (493), peripheral arterial occlusive disease (444), and atrial fibrillation (427.31).

### 2.5 Statistical analysis

The *χ*^2^ test or unpaired *t* test was used to compare data between the metformin users and nonusers in univariate analysis, where appropriate. The Kaplan-Meier method was used to evaluate the MACE-free rate. Survival analysis (log-rank test in univariate analysis and a time-dependent Cox proportional hazards model in multivariate analysis) was used to examine the effect of metformin on the incidence of MACEs. Hazard ratios (HRs) and their 95% confidence intervals (CIs) were calculated. In the secondary analysis, in-hospital death was considered as a competing risk. The significance level of this study was set at 0.05.

## 3 Results

### 3.1 Participants

From 1999 to 2010, 474,410 patients were enrolled. All patients were newly diagnosed with type 2 DM, were not using insulin or any oral antidiabetic medication other than metformin, were older than 18 years, had survived at least 1 month from the diagnosis of DM, and had not been admitted to an intensive care unit within the first month of their DM diagnosis. The mean follow-up duration was 5.86 years. Within the first year of the diagnosis of DM, 36,573 patients (7.7%) were prescribed with metformin and 437,410 patients (92.3%, nonuser group, reference) were not. The mean age of the patients was 55.9 ± 16.1 years, and males accounted for 46.2% of the subjects. A 1:4 propensity score-matched subcohort was selected from the reference group. The demographic characteristics, relevant comorbidities, and medications used during the first year of the diagnosis of DM before and after matching are shown in Table 1.

### 3.2 Incidence of MACEs

**Figure 2:**
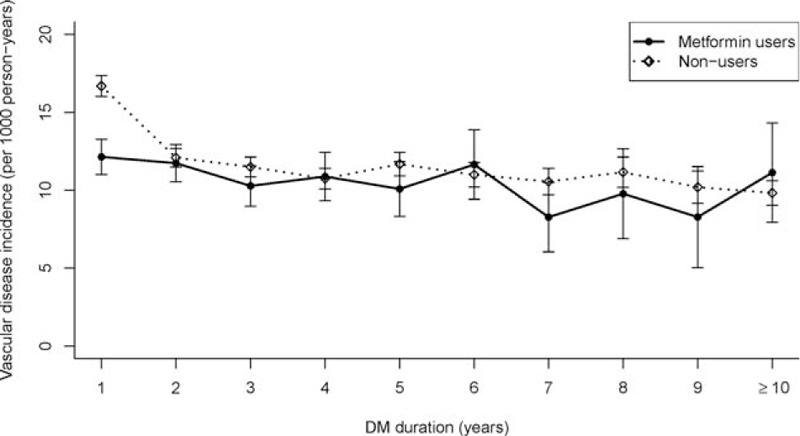
Incidence of major adverse cardiac events with the duration of DM, between users and nonusers of metformin in Taiwan from 1999 to 2010. Patients in the metformin group had a significantly (*P* < .01) lower incidence of MACEs during the first 2 years of DM. DM = diabetes mellitus.

**Figure 3:**
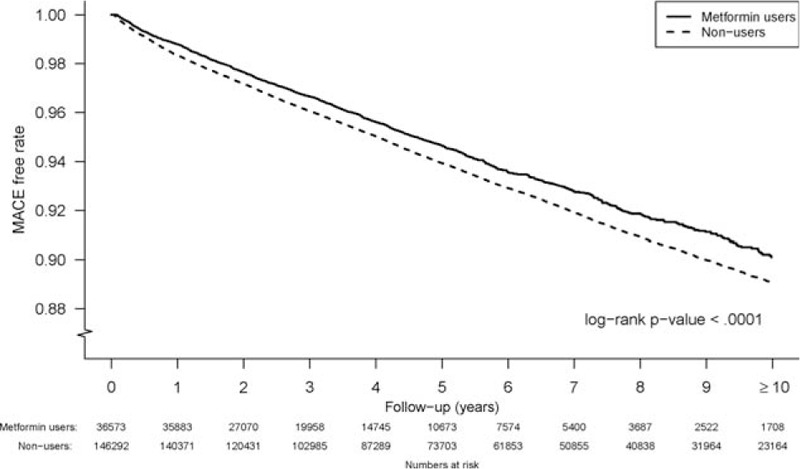
MACE-free survival rate (n = 182,265) for the patients with diabetes with and without metformin use. The metformin users had significantly (*P* < .001) higher MACE-free survival in the first 12 years. MACE = major adverse cardiac event.

The overall MACE rate was 1154.8 per 100,000 person-years, and the MACE rate for the metformin user group was significantly lower than that of the nonuser group (1072.0 vs. 1165.9 per 100,000 person-years, *P* < .001). During years 1 and 2 after the diagnosis of DM, the metformin group had a significantly lower incidence rate of MACEs compared with the lifestyle modification group (Fig. 2). Figure 3 demonstrates the MACE-free survival rates (Kaplan-Meier curves) for the patients with DM who did and did not use metformin. The metformin group had a significantly higher cumulative MACE-free rate during the first 12 years of follow-up than the lifestyle modification group (*P* < .001).

Unadjusted and adjusted HRs of MACEs based on Cox multivariate regression analyses are presented in Table 2. After adjusting for age, sex, hypertension, congestive heart failure, chronic kidney disease, asthma, peripheral arterial occlusive disease, atrial fibrillation, history of MACEs, the use of antihypertensive agents, and statins, the metformin group had a significantly lower MACE rate (HR: 0.83 [95% CI 0.78–0.87], *P* < .001). After considering in-hospital death as a competing risk, the HR of metformin did not change at all.

**Table 2:**
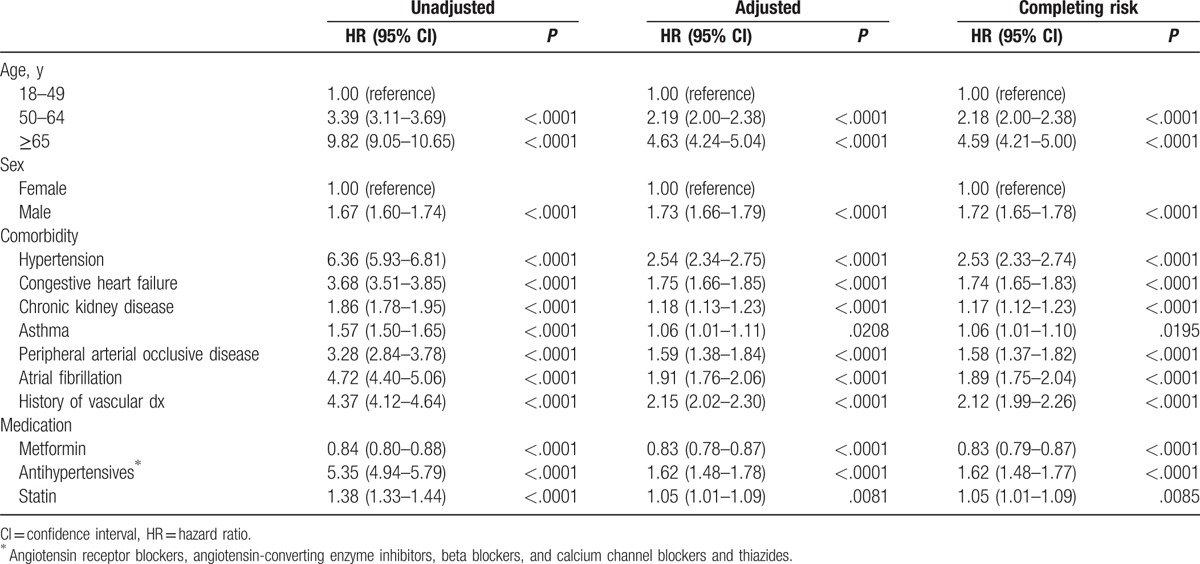
Unadjusted and adjusted HRs of vascular disease among patients with diabetes in Taiwan from 1999 to 2010.

## 4 Discussion

### 4.1 Main results

The main findings of this long-term population-based retrospective cohort study of 474,410 diabetic patients are as follows: in addition to lifestyle modifications, metformin was associated with a decreased risk of MACEs in patients with type 2 diabetes who had not previously used anti-diabetic medications and a metformin-associated protective effect was observed at treatment onset, which remained for at least 2 years while taking metformin. To date, this is the largest cohort observational study comparing the incidence of MACEs between patients with DM who received metformin monotherapy and those with only lifestyle modifications.

### 4.2 Interpretations

In this large population-based cohort study, we found that for patients with newly diagnosed type 2 DM, in addition to lifestyle modifications, metformin monotherapy was associated with fewer MACEs compared to lifestyle modifications alone without any hypoglycemic agents. To the best of our knowledge, this is the largest cohort study comparing MACEs between these2 groups of patients.

Of the enrolled patients, 437,837 with newly diagnosed type 2 DM were not prescribed with any hypoglycemic agents during a mean 5.8 years of follow-up. Considering that the patients were covered by a national medical insurance system, access to medication was not a barrier to any such prescriptions. In guidelines on the management of diabetes, metformin is recommended as the first-line therapy.^[22,23]^ Therefore, these patients may not have been prescribed with metformin owing to good glycemic control with lifestyle modifications, metformin intolerance, or both. After applying the exclusion criteria (Fig. 1), these reasons should be applicable to most of the metformin nonusers.

Lifestyle modifications are advised for all patients with newly diagnosed type 2 DM for glycemic control. Several landmark randomized controlled clinical trials have demonstrated that strict glycemic control reduces the risk of the microvascular and neurological complications associated with diabetes, but not cardiovascular complications. Therefore, strict glycemic control with OHAs is not frequently recommended for these patients.

Metformin has many pleiotropic effects, including decreased hyperglycemia, hypoinsulinemia, higher peripheral muscle glucose uptake, decreased hepatic glyconeogenesis, reduced hypercoagulability, improved lipid profile, and increased nitric oxide-mediated vasodilatation.^[23–26]^ Metformin has also been demonstrated to have cardioprotective effects in patients with myocardial infarction, heart failure, and atrial fibrillation.^[2,13,14,27–33]^ The mechanism of this proposed protective effect is beyond the scope of this study.

The protective effects of metformin seemed to be sustained for only the first 2 years.

This suggests that the early use of metformin in patients with newly diagnosed type 2 DM (i.e., the first 2 years) is associated with more favorable cardiovascular outcomes. The MACE rate after the third year was similar between the metformin and lifestyle change groups. This may be because the severity of hyperglycemia in diabetes progresses with age, and therefore more patients would take hypoglycemic agents as the disease advances with time but were not included in this study. In our cohort, the patients who could sustain lifestyle changes alone for long duration (>2 years) should have had milder hyperglycemia, and we speculate that these patients would be associated with fewer cardiovascular events than those with more severe hyperglycemia who needed OHAs. Therefore, the beneficial effect of metformin is likely to be ameliorated by the severity of hyperglycemia in the metformin monotherapy group.

### 4.3 Limitations

The diagnoses of DM, MACEs, comorbid conditions, and the use of medications were collected from a nationwide registry. Owing to the limitations inherent in an insurance claims database, it is possible that potential errors, bias, and confounding factors may have influenced the completeness and accuracy of the current dataset.

The lack of laboratory information prevented further analysis and validation of the mechanism of our observations. For example, data on hemoglobin A1c were not available in this database. Therefore, we could not evaluate the extent and changes in hyperglycemia. In our study cohort, none of the patients received any OHA other than metformin during follow-up. As the patients did not need to pay for the medications, we speculate that the patients with diabetes without OHAs during follow-up should have had a milder severity of diabetes, and possibly even milder than those with metformin. Although the diagnoses of hypertension and hyperlipidemia were included in the model, blood pressure and lipid profiles between groups were not compared. Most importantly, the adhesion to lifestyle modifications was not measured or monitored in either group. Therefore, the probable effect of lifestyle modifications could not be compared in this study. Furthermore, potential selection bias between the groups could not be analyzed in the current analysis despite the use of propensity score matching. For example, it is possible that physicians tend to prescribe metformin for patients with more advanced hyperglycemia. However, the favorable outcomes associated with metformin in this study suggest that metformin may overcome any adverse selection or bias.

## 5 Conclusion

In this nationwide population-based study, in addition to lifestyle modifications, metformin montherapy was independently associated with a lower risk of MACEs in patients with type 2 DM. The beneficial effects of metformin for patients with pre- or early diabetes may require large, prospective, and randomized trials.
